# Vaccinia virus protein N2 is a nuclear IRF3 inhibitor that promotes virulence

**DOI:** 10.1099/vir.0.054114-0

**Published:** 2013-09

**Authors:** Brian J. Ferguson, Camilla T. O. Benfield, Hongwei Ren, Vivian H. Lee, Gordon L. Frazer, Pavla Strnadova, Rebecca P. Sumner, Geoffrey L. Smith

**Affiliations:** 1 Department of Pathology, University of Cambridge, Tennis Court Road, Cambridge, CB2 1QP, UK; 2 Department of Virology, Faculty of Medicine, Imperial College London, Norfolk Place, London W2 1PG, UK

## Abstract

Vaccinia virus (VACV) expresses many proteins that are non-essential for virus replication but promote virulence by inhibiting components of the host immune response to infection. These immunomodulators include a family of proteins that have, or are predicted to have, a structure related to the B-cell lymphoma (Bcl)-2 protein. Five members of the VACV Bcl-2 family (N1, B14, A52, F1 and K7) have had their crystal structure solved, others have been characterized and a function assigned (C6, A46), and others are predicted to be Bcl-2 proteins but are uncharacterized hitherto (N2, B22, C1). Data presented here show that N2 is a nuclear protein that is expressed early during infection and inhibits the activation of interferon regulatory factor (IRF)3. Consistent with its nuclear localization, N2 inhibits IRF3 downstream of the TANK-binding kinase (TBK)-1 and after IRF3 translocation into the nucleus. A mutant VACV strain Western Reserve lacking the *N2L* gene (vΔN2) showed normal replication and spread in cultured cells compared to wild-type parental (vN2) and revertant (vN2-rev) viruses, but was attenuated in two murine models of infection. After intranasal infection, the vΔN2 mutant induced lower weight loss and signs of illness, and virus was cleared more rapidly from the infected tissue. In the intradermal model of infection, vΔN2 induced smaller lesions that were resolved more rapidly. In summary, the N2 protein is an intracellular virulence factor that inhibits IRF3 activity in the nucleus.

## Introduction

Viruses and their hosts have co-evolved and the pressure of infection by viruses has driven the evolution of the immune system. Equally, the pressure of the immune system has driven the evolution of viruses. For example, mammalian viruses display a wide array of proteins that antagonize the interferon (IFN) system (Randall & Goodbourn, 2008) and each mammalian virus probably has at least one mechanism of evading or blocking the function of IFNs. The range of viral defences against the immune system is very wide and this is particularly evident in large DNA viruses which, in part due to their greater coding capacity, express scores of proteins that target the immune response to infection. Poxviruses are one group of large DNA viruses that encode many such proteins (Seet *et al.*, 2003). Poxviruses replicate in the cytoplasm and include variola virus (VARV), the cause of the disease smallpox, and vaccinia virus (VACV), the vaccine used to eradicate smallpox (Moss, 2007).

After smallpox was eradicated research with VACV might have declined, but paradoxically, research with VACV and other poxviruses has remained as intense as ever. This is partly due to the development of VACV as an expression vector (Mackett *et al.*, 1982; Panicali & Paoletti, 1982) that has been used widely as a laboratory tool for the expression of genes in mammalian cells (Mackett & Smith, 1986; Moss, 1996) and has, for instance, led to an increased understanding of which virus antigens are recognized by cytotoxic T cells (Yewdell *et al.*, 1985; Bennink *et al.*, 1986; Osman *et al.*, 1999) and the identification of the HIV co-receptor CXCR4 (Feng *et al.*, 1996). Enduring interest in VACV also derives from the potential of these viruses as live recombinant viruses (Panicali *et al.*, 1983; Smith *et al.*, 1983a, b), from their utility for studying how viruses exploit cell biology for transmission within and between cells (Cudmore *et al.*, 1995; Doceul *et al.*, 2010), and as a means to study virus–host interactions and discover fundamental aspects of the immune system (Alcamí & Smith, 1996).

VACV expresses many different proteins that combat the immune system. These include proteins that function outside the infected cell to bind and intercept IFNs, cytokines, complement factors and chemokines, proteins on the cell surface that bind such factors or alter the recognition of infected cells by cells of the immune system to the virus’ advantage, and proteins that function intracellularly to block the activation of the pro-inflammatory signalling cascades or apoptosis (for reviews see Smith *et al.*, 1997; Seet *et al.*, 2003; Bowie & Unterholzner, 2008; Bahar *et al.*, 2011). VACV also creates an immunosuppressive environment by the expression of an enzyme that synthesizes immunosuppressive steroid hormones (Moore & Smith, 1992; Reading *et al.*, 2003). One group of intracellular proteins belongs to the B-cell lymphoma (Bcl)-2 family and VACV is predicted to encode ten such proteins (Graham *et al.*, 2008; González & Esteban, 2010). Five of these, N1 (Aoyagi *et al.*, 2007; Cooray *et al.*, 2007), B14 (Graham *et al.*, 2008), A52 (Graham *et al.*, 2008), F1 (Kvansakul *et al.*, 2008), and K7 (Kalverda *et al.*, 2009; Oda *et al.*, 2009) have had their structure solved by X-ray crystallography or nuclear magnetic resonance. N1 (Kotwal *et al.*, 1989; Bartlett *et al.*, 2002), B14 (Chen *et al.*, 2006), A52 (Harte *et al.*, 2003) and K7 (Benfield *et al.*, 2013) each contribute to virulence and all five proteins have had at least one binding partner in host cells identified (Harte *et al.*, 2003; DiPerna *et al.*, 2004; Stewart *et al.*, 2005; Wasilenko *et al.*, 2005; Postigo *et al.*, 2006; Cooray *et al.*, 2007; Chen *et al.*, 2008; Campbell *et al.*, 2010; Maluquer de Motes *et al.*, 2011). Another two VACV Bcl-2 proteins, C6 and A46, have been shown to contribute to virulence (Stack *et al.*, 2005; Unterholzner *et al.*, 2011; Sumner *et al.*, 2013) and have binding partners and a mechanism of action defined (Bowie *et al.*, 2000; Stack *et al.*, 2005; Unterholzner *et al.*, 2011). Lastly, N2, B22 and C1 are other predicted VACV Bcl-2 proteins that are uncharacterized hitherto. This paper concerns the N2 protein.

There is little literature on the *N2L* gene or its encoded protein. Early studies noted this gene was transcribed early during infection (Morgan & Roberts, 1984) and that a mutation in its 5′ non-coding region affected the sensitivity of VACV to the inhibitor of RNA polymerase II, alpha-amanitin (Tamin *et al.*, 1988, 1991) but this observation remains unexplained. The protein has 175 aa residues with a predicted mass of 20.8 kDa and an isoelectric point (pI) of 6.51 (www.poxvirus.org). A yeast-2 hybrid screen reported that N2 bound importin alpha 1, valosin containing protein (p97)/p47 complex interacting protein 1 (VCPIP1) and phospholipid scramblase 4 (PLSCR4) (Zhang *et al.*, 2009), but the biological relevance of these interactions is unknown. Subsequently, N2 was predicted to be a member of the Bcl-2 family and, given the emerging functions of other members of this family, it was hypothesized that N2 might be an intracellular immunomodulator (González & Esteban, 2010). This paper addresses this hypothesis and shows that N2 is an intracellular protein that inhibits activation of interferon regulatory factor (IRF)3 within the nucleus, downstream of IRF3 phosphorylation by TRAF family member-associated NF-kappa-B activator (TANK) binding kinase (TBK)-1, and contributes to virus virulence.

## Results

### Bioinformatic analysis of protein N2

A bioinformatics analysis of the predicted N2 protein showed that there are very highly conserved orthologues (>95 % aa identity) of the VACV strain Western Reserve N2 protein in several other sequenced VACV strains such as Copenhagen (Goebel *et al.*, 1990), 3737, Acam-2000, Duke, Lister and derivatives such as LC16m8 (Morikawa *et al.*, 2005) and rabbitpox virus (www.poxvirus.org). N2 orthologues are also present in the majority of other sequenced orthopoxviruses such as cowpox virus (strains Brighton Red, GRI and Ger-91) VARV (in more than 50 strains) (Esposito *et al.*, 2006), ectromelia virus, horsepox virus and monkeypox virus. In VACV strains MVA (Antoine *et al.*, 1998) and Acam-3000 the N2 protein is 5 aa shorter due to the deletion of aa residues 31–35 and the gene is disrupted in camelpox virus strains CMS (Gubser & Smith, 2002) and M96 (Afonso *et al.*, 2002) and in taterapox virus (Esposito *et al.*, 2006). The conservation in many orthopoxviruses suggests an important function for N2.

**Fig. 1.**
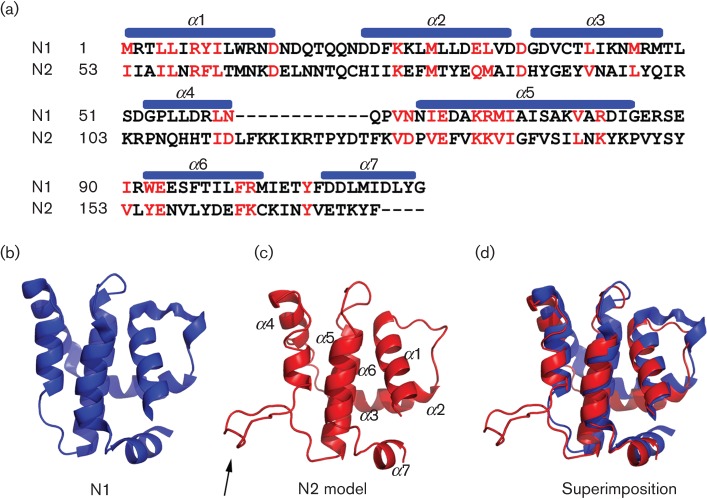
N2 has a predicted Bcl-2-like structure. (a) Sequence alignment of VACV WR protein N2 (residues 53–175) with N1 (residues 1–116). The conserved residues, of which the majority are hydrophobic core residues that stabilize the helical fold, are highlighted in red. Positions of the alpha helices are noted by blue bars above the N1 sequence. (b) The N1 crystal structure (Protein Database (PDB) ID: 2UXE) exhibiting the Bcl-2-like fold. (c) A model of the N2 tertiary structure based on the alignment in (a) with the extra loop in N2 indicated with an arrow. (d) Superimposition of (b) and (c).

A comparison of the N2 sequence with other proteins showed that N2 is most closely related to the VACV N1 protein with which it shares 14 % aa identity over the regions that align with N1, or 9 % identity over its entire length. This suggests there may have been an ancient gene duplication event subsequent to the acquisition of a Bcl-2-like gene by an ancestral poxvirus. Alignment of the N1 and N2 aa sequences showed that N2 has an N-terminal extension of 52 aa and an internal insertion of 12 aa relative to N1 (Fig. 1a). Modelling the N2 sequence (aa residues 51–175) onto the N1 crystal structure using Modeller, produced a good match with the alpha-helical fold of N1 (Fig. 1b–d) with N2 predicted to have a flexible loop between helices 4 and 5 (Fig. 1c, black arrow). Note that the N-terminal extension of N2 is excluded from the alignment and structural prediction because programme DISOPRED2 predicts it to be an unstructured, flexible region of the N2 polypeptide.

### N2 is expressed early during infection and is located in the nucleus

**Fig. 2.**
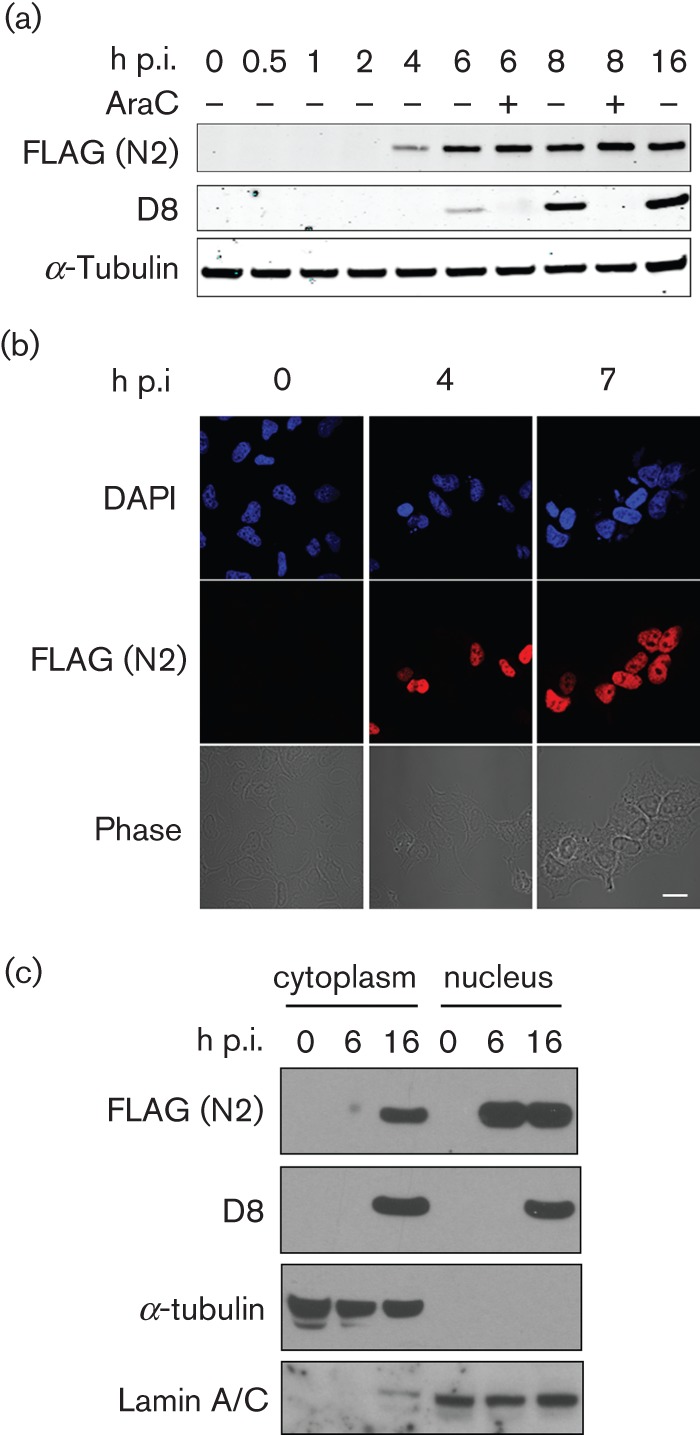
N2 has early expression kinetics and is localized to the nucleus. (a) Time-course of vN2-TAP protein expression in BSC-1 cells. Cells were infected at 10 p.f.u. per cell for the indicated times, with or without araC (40 µg ml^−1^), lysed and the proteins were analysed by immunoblotting with the indicated antibodies. (b) Immunofluorescence detection of N2-TAP following infection. HeLa cells were infected with vN2-TAP at 5 p.f.u. per cell and fixed at the indicated times p.i. Fixed cells were then stained with mouse anti-FLAG antibody, counterstained with DAPI and visualized by confocal fluorescence microscopy. Scale bar, 5 µm. (c) Cells infected with vN2-TAP at 10 p.f.u. per cell were lysed at the indicated times, separated into cytoplasmic and nuclear fractions and analysed by SDS-PAGE and immunoblotting with the indicated antibodies.

To characterize the N2 protein and its expression kinetics and subcellular localization, a recombinant VACV (vN2-TAP) was constructed by transient dominant selection (Falkner & Moss, 1990) (Methods). This virus was constructed from a deletion mutant virus lacking the *N2L* gene (vΔN2), see below. The vN2-TAP virus was engineered to express the N2 protein tagged at its C terminus with a tandem affinity purification (TAP)-tag composed of STREP and FLAG epitope tags (Gloeckner *et al.*, 2007) and was expressed from the *N2L* gene promoter at its natural locus. Cells infected with vN2-TAP were harvested at different times post-infection (p.i.) and extracts of these cells were analysed by SDS-PAGE and immunoblotting with anti-FLAG, anti-α-tubulin and anti-VACV protein D8 antibodies (Abs) (Fig. 2a). This showed that N2 was expressed by 4 h p.i. and in the presence of cytosine arabinoside (araC), an inhibitor of virus DNA replication and thereby intermediate and late virus gene expression. In contrast, D8 expression was inhibited by araC, a characteristic of a late virus protein, and consistent with the known expression kinetics of D8 (Niles & Seto, 1988).

The subcellular localization of N2 was addressed by immunofluorescence (IF) and by cell fractionation. HeLa cells infected with vN2-TAP were processed for IF at 4 and 7 h p.i. and stained with anti-FLAG mAb (Fig. 2b). This showed a strong nuclear fluorescence for N2-TAP that was also seen after transfection of a plasmid expressing TAP-N2 (pTAP-N2) from a human cytomegalovirus (HCMV) immediate early promoter into uninfected HeLa cells, and in a cell line stably expressing this plasmid, TrexTAP-N2 (Fig S1 and Fig. 7c). Parallel protein localization studies using biochemical fractionation of vN2-TAP-infected cells showed that the N2 protein was located in the nucleus at 6 h p.i., although some cytoplasmic N2 was seen at 16 h p.i. (Fig. 2c). The validity of the fractionation was confirmed by immunoblotting with anti-α-tubulin and anti-lamin A/C mAbs.

### N2 is non-essential for virus replication in cell culture

**Fig. 3.**
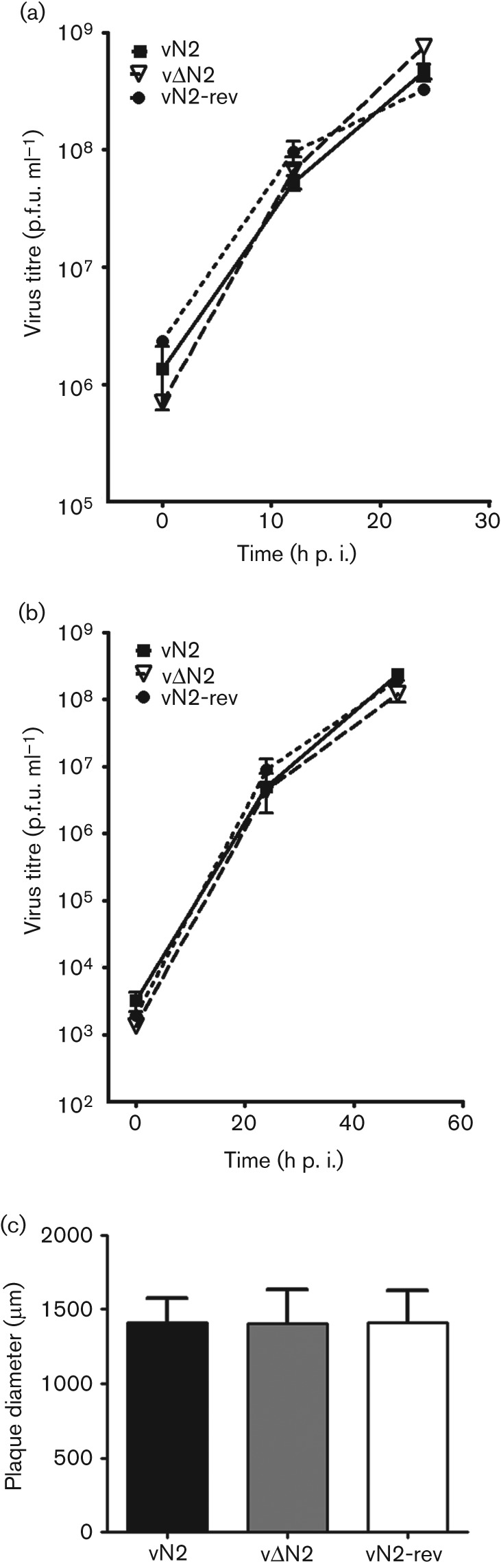
N2 is non-essential for virus replication and spread in culture. BSC-1 cells were infected with the indicated viruses at (a) 10 or (b) 0.01 p.f.u./cell. Cells were harvested at the indicated times and virus infectivity was titrated by plaque assay on BSC-1 cells. (c) BSC-1 cells were infected with the indicated viruses and 72 h p.i the diameter of 30 plaques were measured for each virus. Data are expressed as the mean±sd plaque diameter (*μ*m).

The contribution of the N2 protein to virus replication was assessed next using a recombinant VACV (vΔN2) in which the N2 open reading frame (ORF) was deleted by transient dominant selection (Methods) (Falkner & Moss, 1990). A plaque purified wild-type virus (vN2) was isolated from the same intermediate virus and a revertant virus (vN2-rev) was constructed from the deletion mutant by reinsertion of the *N2L* gene at its natural locus. Analysis of the *N2L* locus of these viruses and vN2-TAP by PCR using primers that bind to the *N2L* gene flanking regions confirmed that the genomes had the structures predicted (Fig. S2). The replication kinetics of vN2, vΔN2 and vN2-rev were compared in cell culture and shown to be indistinguishable for each virus after either high or low m.o.i. (Fig. 3a, b). The spread of these viruses from cell-to-cell was assessed by measuring the plaque size formed on BSC-1 cells and also was indistinguishable (Fig. 3c). Collectively, these analyses indicate that virus replication and spread in cultured cells are not affected by the N2 protein.

### N2 is a virulence factor

**Fig. 4.**
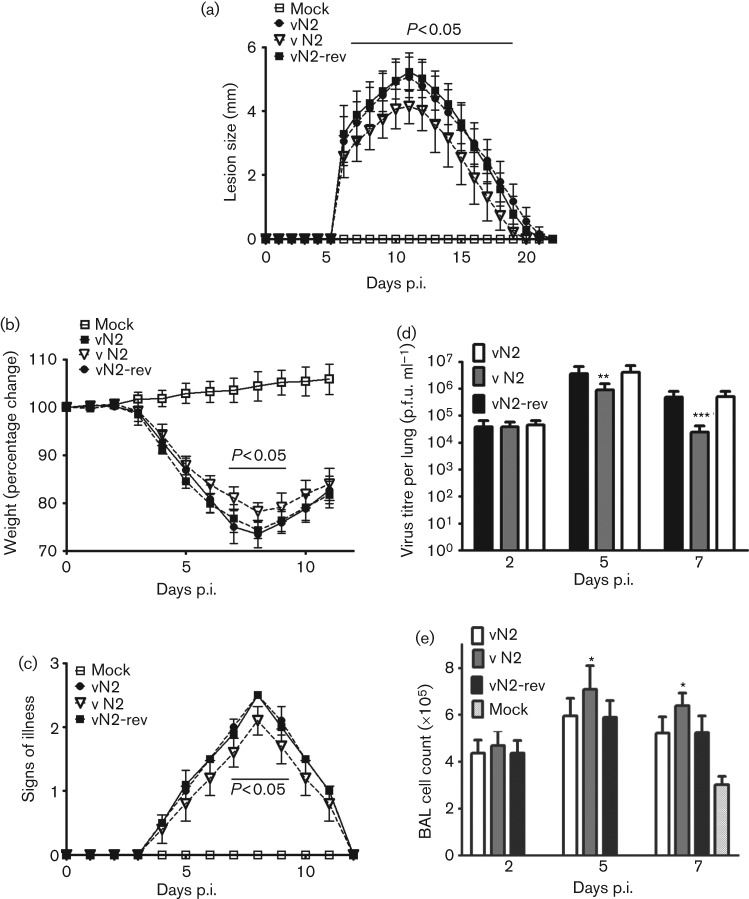
N2 contributes to VACV virulence in mice. (a) Groups of five C57/B6 mice were infected i.d. in the ear pinnae with the indicated viruses and lesion sizes were measured daily. Data are presented as the mean lesion size±SEM (b) Groups of five BALB/c mice were infected i.n. with the indicated viruses and weights and signs of illness were measured daily. Data are presented as the mean weights as a percentage of the mean weight of the same group on day zero. (c) Signs of illness (Alcami & Smith, 1992) as monitored in the same mice as in (b). (a) – (c) Days on which values for vΔN2 were statistically different from both vN2 and vN2-rev are marked by the horizontal bar. (d) Groups of five BALB/c mice were infected i.n. with the indicated viruses, sacrificed at the indicated days p.i. and virus titres from the lungs were measured by plaque assay on BSC-1 cells. Data are mean titre±sd, **P<0.01, ***P<0.001. (e) Groups of five BALB/c mice were infected i.n. with the indicated viruses, sacrificed at the indicated days p.i. and the total number of cells in the BAL fluid were counted. Data are presented as mean cell numbers±sd, *P<0.05.

There are many instances in which a VACV protein is not required for replication or spread in cell culture but yet affects the outcome of infection *in vivo* (Alcamí & Smith, 1992; Moore & Smith, 1992; Symons *et al.*, 1995; Bartlett *et al.*, 2002; Harte *et al.*, 2003; Stack *et al.*, 2005; Chen *et al.*, 2006; Fahy *et al.*, 2008; Unterholzner *et al.*, 2011; Ember *et al.*, 2012; Benfield *et al.*, 2013; Mansur *et al.*, 2013). Therefore, the virulence of vΔN2 was assessed using two murine models. The intranasal (i.n.) model results in systemic infection and virus virulence is assessed by weight loss and signs of illness (Williamson *et al.*, 1990; Alcamí & Smith, 1992). In the intradermal (i.d.) model the infection remains localized around the site of injection and virulence is assessed by lesion size and timing of healing (Tscharke & Smith, 1999). In the i.d. model the lesion caused by infection with vΔN2 was smaller and healed more quickly than those caused by both control viruses with statistically significant differences between 7 and 21 days p.i. (Fig. 4a). In the i.n. model infection with vΔN2 induced less weight loss (Fig. 4b) and fewer signs of illness (Fig. 4c) than control viruses and significant differences in weights were seen on days 7–9 p.i. Measurement of titres of infectious virus in the lungs of infected animals showed that although all viruses replicated to the same extent initially (day two), subsequently (days five and seven), the titres of virus in vΔN2-infected animals were lower than controls (Fig. 4d), implying more rapid clearance. These lower virus titres at days 5 and 7 p.i. were accompanied by increased cell numbers in broncho-alveolar lavage (BAL) fluid, consistent with a stronger immune response to vΔN2 (Fig. 4e). Collectively, these data indicate that the N2 protein is a virulence factor affecting the outcome of infection in both i.d. and i.n. models of infection.

**Fig. 5.**
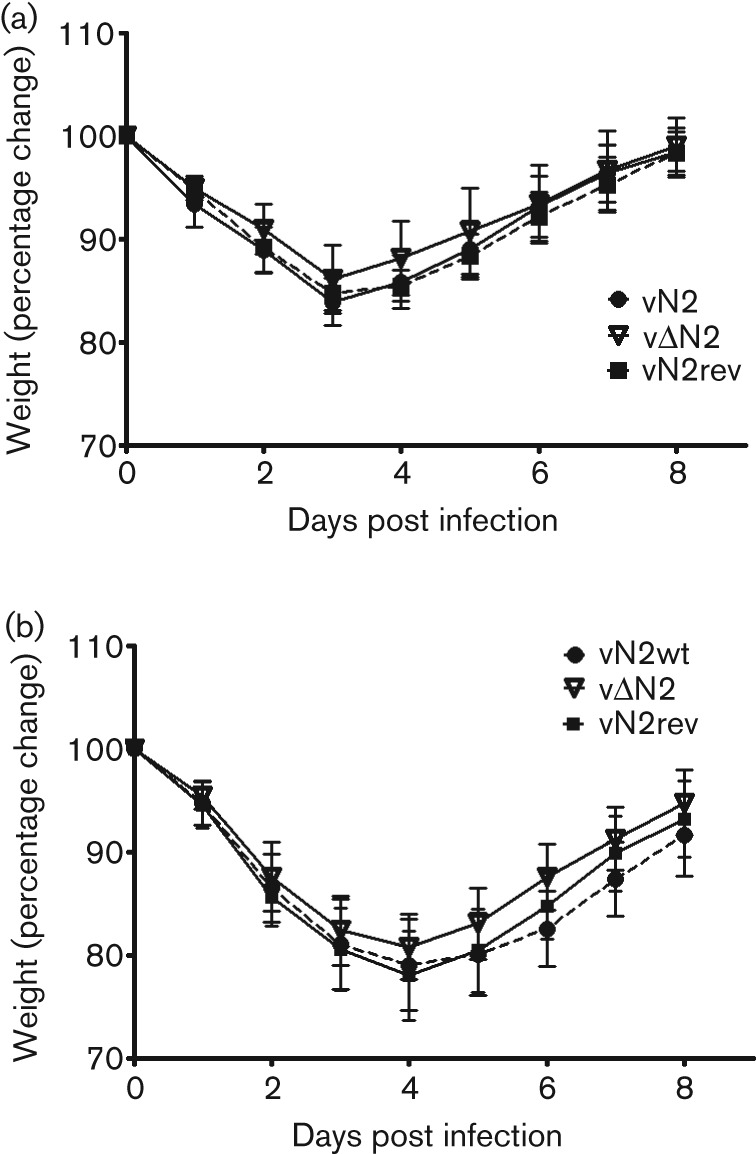
Loss of N2 does not increase virus immunogenicity. Groups of 5 (a) C57/B6 mice or (b) BALB/c mice were infected i.d. or i.n. respectively with the indicated viruses. After 4-6 weeks mice were challenged with a lethal dose of VACV (5×10^6^ p.f.u of VACV WR) and weight change was monitored. Data are expressed as the percentage±sd of the mean weight of the same group of animals on day zero.

The potency of vΔN2 as a vaccine was assessed by infecting mice with vΔN2 or control viruses via either the i.d. or i.n. routes, and then challenging the animals (28 or 42 days later respectively) with a lethal dose of wild-type VACV strain WR. It was found that the protection afforded by vΔN2 against challenge was indistinguishable from control viruses in both infection models (Fig. 5) indicating that the deletion mutant was not functionally more immunogenic in the assay used. This result contrasts with that for another VACV virulence factor (C6) in which the deletion mutant was more immunogenic despite being attenuated (Sumner *et al.*, 2013). A C6 deletion mutant in VACV strain MVA was also more immunogenic (García-Arriaza *et al.*, 2011).

### N2 inhibits activation of IFNβ promoter by inhibiting IRF3 activation

**Fig. 6.**
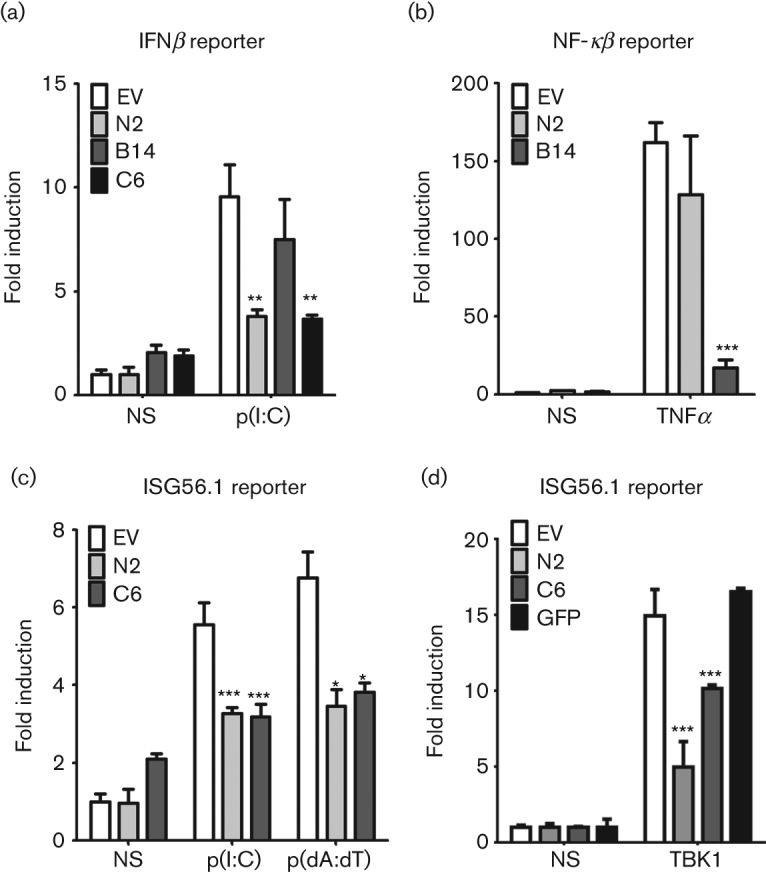
N2 inhibits IRF3 reporter activity. HEK293 cells were co-transfected with the indicated reporter and expression plasmids, and 24 h later were stimulated for 6 h with (a) and (c) poly(I : C) or poly(dA:dT) or (b) TNFα. Cells were then lysed and firefly luciferase activity was measured and normalized to renilla luciferase activity. Data are from triplicate samples from one representative experiment of at least 3, presented as mean±sd *P<0.05; **P<0.01, ***P<0.001 relative to empty vector (EV).

The mechanism of action of N2 was addressed next. The knowledge that several members of the VACV Bcl-2 protein are inhibitors of intracellular signalling pathways (Introduction), together with the observations that vΔN2 had reduced virulence and yet could induce recruitment of more cells into infected tissue, suggested that the N2 protein might also modulate innate immune signalling pathways and this hypothesis was tested initially using luciferase reporter assays. A reporter plasmid in which luciferase was driven by an IFNβ promoter was transfected into HEK293T cells and luciferase activity was measured after stimulation with poly(I : C) in the absence (empty vector) or presence of the N2 protein. This showed that N2 inhibited activation of the IFNβ promoter (Fig. 6a). In parallel, the VACV C6 protein did, and the B14 protein did not, inhibit this promoter, consistent with the known functions of these proteins (Chen *et al.*, 2008; Unterholzner *et al.*, 2011).

The IFNβ promoter contains binding sites for NF-κB and AP-1 as well as IRF3, and therefore N2 might have diminished activation of the IFNβ promoter by inhibiting any one, or more than one, of these transcription factors. To investigate this, a reporter plasmid containing luciferase driven by an NF-κB specific promoter was utilized. N2 was unable to inhibit NF-κB activation stimulated by tumour necrosis factor (TNF)α, whereas a known inhibitor of this pathway, B14 (Chen *et al.*, 2008), was inhibitory (Fig. 6b). In addition, nuclear translocation of p65 was also measured by immunofluorescence and found to be unaffected by the presence of N2 (Fig. S2). In contrast, N2 inhibited expression from an IRF3-specific reporter plasmid (ISG56.1) in response to poly(I : C) or poly(dA:dT), as did C6, a known inhibitor of this pathway (Fig. 6c).

**Fig. 7.**
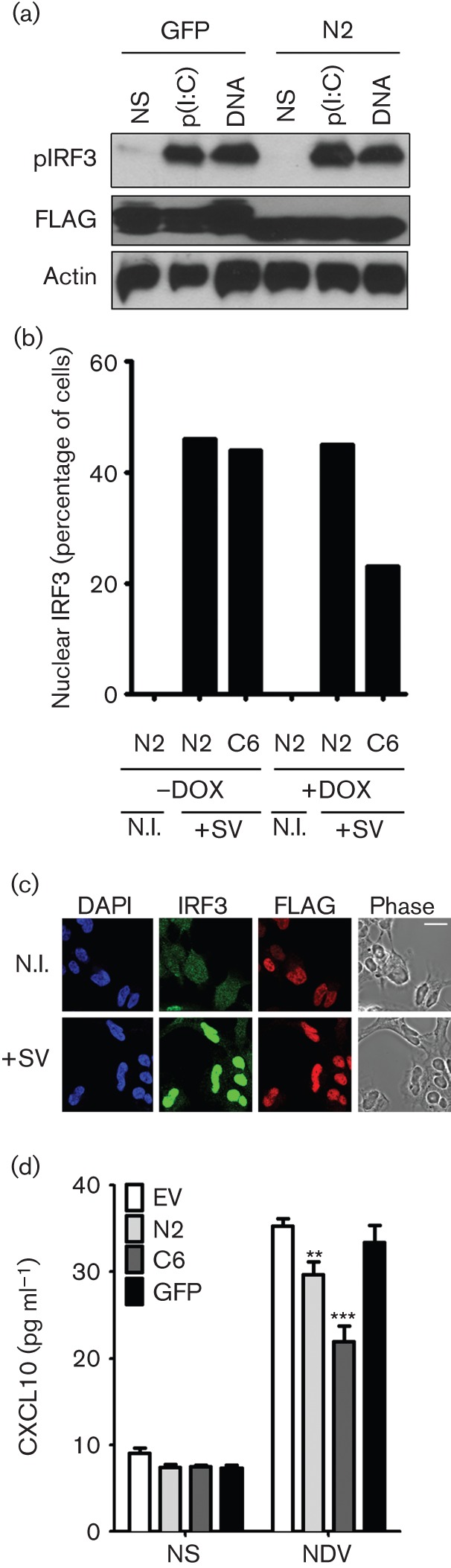
N2 inhibits IRF3 activity after IRF3 phosphorylation and nuclear translocation. (a) Trex STING-TAP cells (see Methods) were transfected with GFP-FLAG or TAP-N2 and 24 h later were stimulated by transfection with poly(I : C) or immunostimulatory DNA. 3 hours later cells were lysed and immunoblotted with the indicated antibodies. (b) TrexTAP-N2 (N2) or TrexTAP-C6 (C6) cells were left resting or induced with 2 µg doxycycline (DOX) ml^−1^ then not infected (N.I.) or infected with Sendai virus (SV) for 6 h before being fixed and immunostained with FLAG and IRF3 antibodies. Cells with nuclear IRF3 were counted under a fluorescence microscope and are presented as a percentage of a total of least 50 cells from random fields of view. Data are representative of three experiments. (c) Confocal images of IRF3 and TAP-N2 (immunostained with a FLAG mAb) in TrexTAP-N2 cells following addition of 1 µg DOX ml^−1^ and infection with SV. Scale bar, 5 µm. (d) HEK293 cells were transfected with the indicated plasmids and then infected with the attenuated NDV vaccine strain clone 30 for 16 h. CXCL10 in the cell supernatants was measured by ELISA (*n* = 3, mean±SD, **P<0.01, ***P<0.001).

To examine where N2 was acting in the pathway leading from RNA or DNA stimulation to IFNβ promoter activation, the overexpression of TIR-domain-containing adaptor-inducing interferon-β (TRIF) and TANK binding kinase 1 (TBK1) was used to stimulate this pathway. In both cases N2 was inhibitory indicating that it acts downstream of TBK1 activity (Fig. S3). This finding was confirmed by showing that N2 could also inhibit TBK1-induced ISG56.1 promoter activity (Fig. 6d). Since one of the major functions of TBK-1 in the innate immune response is to phosphorylate IRF3 on serines 392 and 396 leading to IRF3 dimerization and translocation into the nucleus (Fitzgerald *et al.*, 2003), N2 might inhibit IRF3 activity by preventing IRF3 phosphorylation or translocation into the nucleus, or by affecting a downstream step such as assembly or function of the transcriptional complex within the nucleus. Immunoblotting showed that IRF3 phosphorylation induced by poly I : C or DNA stimulation was unaltered in the presence of N2 (Fig. 7a) and immunofluorescence showed that in the presence or absence of N2 IRF3 still translocated into the nucleus after stimulation (Fig. 7b, c). However, N2 reduced the expression of chemokine CXCL10, which is regulated by an IRF3-responsive promoter, from cells infected with Newcastle disease virus (NDV) (Fig. 7d), providing further evidence of its ability to inhibit the IRF3-dependent innate immune response to virus infection. Overall, these data show that VACV N2 can function in the nucleus to disrupt the activity of IRF3 following the activation of this key innate immune transcription factor in the cytoplasm and its subsequent nuclear translocation.

## Discussion

*N2L* is a highly conserved gene in VACV and several other orthopoxviruses, and is a member of the VACV Bcl-2 family (González & Esteban, 2010). However, prior to this study little was known about the function of N2. In this paper the N2 protein from VACV strain WR is shown to be a virulence factor that is predominantly present in the nucleus of infected cells where it inhibits IRF3 activity. N2 is the fourth VACV protein that blocks IRF3 function, the others being A46, K7 and C6, but despite the presence of these other inhibitors, the deletion of N2 caused an overt *in vivo* phenotype, showing that these proteins have non-redundant functions. A46 binds TRIF (Stack *et al.*, 2005), K7 binds to DDX3 (Schröder *et al.*, 2008) and C6 binds to the TBK-1 and inhibitor of kappa B kinase ϵ (IKKϵ) adaptor proteins TANK, NAK-associated protein 1 (NAP1) and similar to NAP1 TBK1 adaptor (SINTBAD) (Unterholzner *et al.*, 2011), and so all three VACV proteins can block IRF3 activation in the cytoplasm. In contrast, N2 functions further downstream in the signalling pathway, following the translocation of IRF3 into the nucleus, but still inhibits transcriptional activity (from the IFNβ or ISG56.1 promoters).

N2 is most similar to N1 in aa sequence, and structure prediction analysis using hidden Markov models indicates that this protein has a very high probability of maintaining the Bcl-2-like fold exhibited by N1 and eight other VACV proteins (Graham *et al.*, 2008; González & Esteban, 2010). Interestingly though, these proteins show very low levels of primary sequence identity (N2 has only 14 % identity to N1 over the aligned regions of these polypeptides) and in general this family presents an excellent example of how protein structure can be conserved in the absence of primary sequence conservation. It is also intriguing how the function of proteins in this family, although diverse in mechanism, is so far restricted to inhibition of host-defence signalling pathways. Hence, in this family, structure and function are correlated, but sequence and mechanism are variable.

This study has shown that N2 functions in the nucleus to inhibit IRF3, although the precise molecular mechanism of action remains unknown. This is reminiscent of the measles virus C protein (Sparrer *et al.*, 2012) and the Nipah virus W protein (Shaw *et al.*, 2005) both of which can also inhibit nuclear IRF3 activity by unknown mechanisms. The only other functional information about the N2 protein comes from a yeast-2 hybrid screen (Zhang *et al.*, 2009) which suggested that N2 could bind directly to importin alpha 1, VCPIP1 and PLSCR4, although it is currently unclear how these putative interaction partners may contribute to the function of N2 described here. It may be the case that N2 binds importin alpha 1 as a mechanism to enter the nucleus, although a classical nuclear localization sequence could not be identified in its primary sequence. Further work is therefore required to understand how N2 functions to inhibit IRF3 activity.

N2 represents another member of the formidable arsenal of VACV proteins that contributes to defence against the IFN system. As well as the other inhibitors of IRF3 activation, VACV encodes ten intracellular proteins that inhibit activation of NF-κB and thereby contribute to the suppression of the IFN-β promoter activation (Ember *et al.*, 2012; Mansur *et al.*, 2013). If IFNs are produced in response to VACV infection, the virus prevents type I or type II (IFN-γ) from reaching its receptors on cells by secreting soluble IFN binding proteins B8 (Alcamí & Smith, 1995; Mossman *et al.*, 1995) and B18 (Colamonici *et al.*, 1995; Symons *et al.*, 1995), which capture IFNs in solution. The B18 protein also has the interesting property of being able to bind to the surface of both infected and uninfected calls and capture IFNs on the cell surface too (Alcamí *et al.*, 2000). If some IFN is produced and escapes capture by B8 or B18, VACV encodes a tyrosine phosphatase called vH1 that is packaged into virions and delivered into cells with the incoming virion to dephosphorylate the signal transducer and activator of transcription (STAT)1 and STAT2 proteins and so prevent activation of the Janus kinase (JAK)/STAT pathway leading to induction of IFN stimulated genes (ISGs) (Najarro *et al.*, 2001). Finally, if some ISGs are expressed VACV neutralizes their action by the expression of protein E3 and K3. E3 binds dsRNA and thereby blocks activation of IFN-induced antiviral proteins such as protein kinase R or 2′-5′-oligoadenylate synthetase, which require dsRNA for their activation (Chang *et al.*, 1992). K3 functions to prevent phosphorylation of eukaryotic initiation factor 2a (eIF2a) by PKR (Davies *et al.*, 1992). It does this by molecular mimicry and shares sequence similarity to the N-terminal domain of eIF2α.

A comparison of the contribution of N2 to VACV virulence and immunogenicity with the other VACV inhibitors of IRF3 activation is interesting. Deletion of C6, K7 and N2 each reduced virulence in both the i.d. and i.n. models (Unterholzner *et al.*, 2011; Benfield *et al.*, 2013) and loss of A46 caused a modest attenuation in the i.n. model and the effect in the i.d. model is unknown (Stack *et al.*, 2005). The attenuation resulting from loss of N2 was milder in the i.n. model than that resulting from loss of K7 or C6. However, in the i.d. model vΔN2 had a more pronounced attenuation than seen after i.n. infection. It has been noted previously that the loss of a specific VACV immunomodulator may give a phenotype in either, both or neither model (Tscharke *et al.*, 2002). Intriguingly, the loss of C6 enhanced the immunogenicity of VACV strains WR and MVA (García-Arriaza *et al.*, 2011; Sumner *et al.*, 2013), but there was no alteration in immunogenicity induced by loss of N2. This might suggest that C6 and/or N2 have additional, differing functions, and a comparison of the innate immune response to infection by these viruses may shed light on factors inducing a strong immune response to infection.

In summary, N2 is a predicted member of the Bcl-2 family of VACV proteins and like other members of this family is an inhibitor of intracellular innate immune signalling pathways. N2 is expressed in the nucleus where it is able to inhibit IRF3 signalling and, although it does not affect the immunogenicity of VACV strain WR, N2 is a virulence factor, like many other VACV Bcl-2-like proteins. Further studies of N2 will be required to understand its molecular mechanism and may help to uncover further details of how IRF3 functions in the nucleus.

## Methods

### 

#### Plasmid vectors for expression in mammalian cells.

The *N2L* ORF was synthesized as a codon-optimized allele for mammalian cellular expression (Life Technologies) and cloned into pCDNA4-T/O vector modified to express an N-terminal TAP-tag in-frame with the *N2L* gene. This plasmid, pTAP-N2, was used for all subsequent assays in mammalian cells. The plasmids pTAP-C6, pFLAG-B14, pFLAG-GFP and pFLAG-TRIFΔRIP and FLAG-TBK1 were described (Unterholzner *et al.*, 2011).

#### Cell culture.

BSC-1, CV-1, HEK293T and HEK293Trex cells were grown in Dulbecco’s modified Eagle’s medium (Life Technologies) supplemented with 10% FBS (Harlan Laboratories) and penicillin/streptomycin (50 µg ml^−1^; Life Technologies), HEK293Trex cells were also supplemented with blasticidin (100 µg ml^−1^, Life Technologies). Stable cell lines were generated by transfection of pTAP-N2, pTAP-C6 or pSTING-TAP into HEK293Trex cells followed by clonal selection in zeocin (100 µg ml^−1^, Life Technologies) to create TrexTAP-N2, TrexTAP-C6 and TrexSTING-TAP. Expression of N2, C6 or STING in these cells was induced with 2 µg doxycycline ml^−1^ (unless otherwise stated) (Melford Laboratories). HeLa and RK-13 cells were grown in minimum essential medium (Life Technologies) supplemented as above and, for HeLa cells, with non-essential aa (Sigma). All cell lines were maintained at 37 °C in 5 % CO_2_.

#### Recombinant viruses.

An *N2L* deletion VACV (vΔN2), a wild-type virus (vN2) and a revertant virus (vN2-rev) were constructed by transient dominant selection (Falkner & Moss, 1990) as described for the *C4L* gene (Ember *et al.*, 2012). A virus vN2-TAP contained sequences encoding a C-terminal TAP-tag consisting of two StrepII tags and one FLAG tag fused in-frame at the C terminus of the N2 ORF followed by a termination codon (Gloeckner *et al.*, 2007) was also constructed from vΔN2. The genotype of recombinant viruses was determined by PCR together with restriction digestion analysis of genomic viral DNA extracted from sucrose gradient purified virus.

#### Virus growth curves.

BSC-1 cells were infected at 0.01 or 10 p.f.u. per cell and at the indicated times the titre of intracellular virus was determined by plaque assay on BSC-1 cells as described (Ember *et al.*, 2012).

#### Plaque size assay.

BSC-1 cell monolayers were infected for 72 h and well separated plaques were then stained with crystal violet and their sizes (*n* = >50) were measured using Axiovision 4.6 software and a Zeiss Axiovert 200 M microscope as described (Doceul *et al.*, 2010).

#### Reporter assays.

Luciferase reporter gene assays were performed as described (Mansur *et al.*, 2013). HEK293T cells in 96-well plates were transfected with 60 ng per well of firefly luciferase reporter plasmids, 10 ng per well of pTK-Renilla luciferase (pRL-TK, Promega) and the indicated amount of expression vectors with TransitLT1 (Geneflow). IFNβ-promoter luciferase reporter was a gift from T. Taniguchi (University of Tokyo, Japan) and NF-κB-luciferase was from R. Hofmeister (University of Regensburg, Germany). ISG56.1-luciferase was a gift from Ganes Sen (Cleveland Clinic, USA). DNA was kept constant during the transfections by the addition of empty vector control plasmid. Cells were stimulated as indicated in the figures and were harvested in passive lysis buffer (Promega). Data were analysed using MARS data analysis software on a FLUOstar Omega instrument (BMG Labtech). The relative stimulation of reporter-gene expression was calculated by normalizing firefly luciferase activity with renilla luciferase activity. In all cases, data shown are representative from at least three independent experiments. Poly(I : C) (high molecular mass) and poly(dA:dT) were from Invivogen and immunostimulatory DNA was as described (Ferguson *et al.*, 2012).

#### Virulence assays.

Female BALB/c mice (*n* = 5; 6–8 weeks old) were anaesthetized and infected i.n. with 5×10^3^ p.f.u. and monitored as described (Alcamí & Smith, 1992). Female C57BL/6 mice (*n* = 5; 6–8 weeks old) were anaesthetized and infected i.d. with 10^4^ p.f.u. and the lesion size was measured daily with a micrometer as described (Tscharke & Smith, 1999). For challenge experiments, mice that had been infected either i.n. or i.d. (as above) were anaesthetized 4-6 weeks later, challenged i.n. with 5×10^6^ p.f.u. of VACV WR and their weights were measured for 8 days as described (Sumner *et al.*, 2013).

#### Broncheoalvoelar lavage and lung titrations.

These were performed as described (Reading *et al.*, 2003). Briefly, BALB/c mice were infected i.n. with 5×10^3^ p.f.u. and at time indicated were sacrificed, and a polyethylene catheter was inserted into the trachea. The lungs were inflated with 1.5 ml RPMI 1640 medium, which was then removed with a 2 ml syringe. This was repeated twice more and the samples were combined to represent the bronchoaleolar lavage (BAL) fluid. The BAL fluid was centrifuged at 300 ***g*** for 10 min and the cell pellets were suspended in 1 ml medium and viable cells were counted by trypan blue exclusion. The washed lungs were homogenized, and the infectious viral titre was assayed by plaque assay on BSC-1 cells.

#### Bioinformatics.

The homology modelling software MODELLER (Martí-Renom *et al.*, 2000) was used to create 30 potential models of N2 using the N1/N2 sequence alignment, which were analysed using the PROCHECK (Laskowski *et al.*, 1993), WHAT IF (Vriend, 1990), and Verify3D (Lüthy *et al.*, 1992) algorithms to ensure the satisfaction of stereochemical restraints. The model with the lowest energy and fewest spatial violations was selected as the most accurate representation of this domain.

#### Cell fractionation and immunoblotting.

Cells were separated into nuclear and cytoplasmic fractions by swelling in hypotonic buffer, dounce homogenization and centrifugation as described (Fahy *et al.*, 2008). Immunoblotting was performed as described previously (Mansur *et al.*, 2013). Antibodies were from the following sources: mouse anti-FLAG (Sigma), mouse anti-α-tubulin (Upstate Biotech), rabbit anti-pIRF3 (Abcam) and mouse anti-lamin A/C (Abcam). Mouse anti-D8 mAb AB1.1 was described previously (Parkinson & Smith, 1994).

#### Immunofluorescence.

Cells were fixed in 4 % paraformaldehyde, permeabilized in 0.5 % Triton in PBS, pre-incubated for 1 h in blocking buffer (5 % BSA, 0.0 5 % Tween-20 in PBS), stained for 3 h with primary antibody and for 1 h with Alexa488 or Alexa647-labelled secondary antibodies (1 : 500, Life Technologies). Coverslips were mounted in MOWIOL 4-88 (Calbiochem) containing DAPI. Images were taken on an Olympus FV1000 scanning confocal microscope. Primary antibodies used were mouse anti-FLAG (Sigma), rabbit anti-FLAG (Sigma), rabbit anti-IRF3 and mouse anti-p65 (both Santa Cruz Biotechnology). Secondary antibodies used were Alexa Fluor 488–donkey anti-mouse and Alexa Fluor 546–goat anti-rabbit (both Life Technologies).

#### Statistical analyses.

Statistical analysis was carried out using Student’s *t*-test with Welch's correction where necessary.
